# Multi‐Strain Probiotics BLa80, LRa05, and BBr60 Modulate Inflammation, Bile Acids, and Gut Microbiota in Type 2 Diabetes: A Randomized Controlled Trial

**DOI:** 10.1002/fsn3.71735

**Published:** 2026-04-10

**Authors:** Sijia Zhu, Yan Qiao, Wenjing He, Yaojie Xiao, Guiqin Song, Kang Liu, Shuguang Fang

**Affiliations:** ^1^ Cancer Biotherapy Key Laboratory of Nanchong Beijing Anzhen Nanchong Hospital of Capital Medical University and Nanchong Central Hospital, the Second Clinical Medical College of North Sichuan Medical College Nanchong Sichuan China; ^2^ Department of Endocrinology Beijing Anzhen Nanchong Hospital of Capital Medical University and Nanchong Central Hospital, the Second Clinical Medical College of North Sichuan Medical College Nanchong Sichuan China; ^3^ Wecare Probiotics R&D Centers (WPC) Wecare Probiotics Co., Ltd Suzhou Jiangsu China

**Keywords:** bile acid metabolism, gut microbiota, inflammation, probiotics, type 2 diabetes mellitus

## Abstract

To evaluate the effects of a multi‐strain probiotic formula, *Bifidobacterium animalis* subsp. *lactis* BLa80, *Lacticaseibacillus rhamnosus* LRa05, and *Bifidobacterium breve* BBr60, on inflammation, metabolism, and the gut microbiota in patients with type 2 diabetes mellitus (T2DM). In a randomized, double‐blind, placebo‐controlled trial, 80 adults with T2DM received either the probiotic or a placebo in addition to standard hypoglycemic therapy for 12 weeks. We assessed inflammatory cytokines, glycemic indices, serum amino acids, bile acids (BAs), short‐chain fatty acids (SCFAs), and gut microbiota composition. Compared with the placebo group, the probiotic intervention led to a significant reduction in the levels of IL‐17 and TNF‐α (*p < 0.05*), reduced serum concentrations of threonine, isoleucine, and arginine. Additionally, the BA profile was markedly altered, revealing 16 differential metabolites that were associated with pivotal metabolic pathways. SCFA analysis showed higher isobutyric and isovaleric acid after supplementation. The probiotic group also exhibited significant reductions in total glycated hemoglobin (GHb) and fasting plasma glucose (FPG, *p* < 0.05). Microbiome analyses indicated decreased alpha‐diversity and distinct beta‐diversity shifts, including increased Gemmatimonadota and reduced *Clostridium* abundance in the probiotic group. This multi‐strain probiotic modulated inflammatory responses, metabolic profiles, including BA metabolism and SCFAs, and gut microbiota composition in T2DM, supporting its potential as an adjunct to metabolic management.

**Trial Registration:** ClinicalTrials.gov identifier: NCT06440486

## Graphical abstract

This randomized, double‐blind, placebo‐controlled trial investigated the effects of a defined multi‐strain probiotic supplementation in patients with type 2 diabetes mellitus. After 12 weeks of intervention, probiotic intake was associated with selective modulation of gut microbiota composition, alterations in microbial‐related metabolic pathways involving bile acids, short‐chain fatty acids, and amino acids, and attenuation of the pro‐inflammatory milieu. These coordinated changes were accompanied by improvements in glycemic‐related parameters, supporting an interaction among gut microbiota, inflammation, and host metabolism in T2DM.

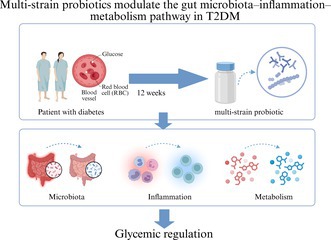

## 1 Introduction

Type 2 diabetes mellitus (T2DM) is the most common form of diabetes, making up over 90% of all diabetes cases globally. It is characterized by insulin resistance or relative insulin deficiency, which together result in impaired glucose utilization and chronic hyperglycemia (Global, Regional, and National Burden of Diabetes From 1990 to 2021, With Projections of Prevalence to 2050: A Systematic Analysis for the Global Burden of Disease Study 2021 2023; Eizirik et al. 2020; Tahoun et al. 2024). The global prevalence of T2DM has increased sharply in recent decades, driven by both genetic predisposition and lifestyle factors such as physical inactivity and high‐calorie diets (Antony and Vijayan 2021). Global diabetes prevalence affected approximately 537 million adults in 2021, as reported by the International Diabetes Federation (IDF). Projections indicate a substantial increase to 643 million by 2030, with a further rise to 783 million anticipated by 2045 a growth rate that exceeds global population trends International Diabetes Federation [IDF]; www.diabetesatlas.org.

Emerging evidence indicates that dysbiosis of the gut microbiota plays a pivotal role in the pathogenesis of T2DM development, primarily through its contribution to insulin resistance and chronic systemic inflammation (Dulai et al. 2024). The gut microbiota is essential for maintaining intestinal barrier integrity, regulating host metabolism, and modulating immune homeostasis (Yoo et al. 2020; Kayama et al. 2020). Disruptions in microbial composition can impair these functions, triggering metabolic disturbances and inflammatory responses that exacerbate glycemic dysregulation (Gill et al. 2022; Liu, Zhang, et al. 2023). Probiotics, characterized as live microbial supplements that impart health advantages when consumed in sufficient quantities, are increasingly regarded as a promising adjunctive therapy for T2DM. Their therapeutic potential is primarily attributed to their capacity to positively reshape the gut microbiota composition. Clinical and experimental studies have demonstrated that probiotic supplementation can improve glycemic control and inflammatory status in T2DM patients by enhancing immune regulation, reducing oxidative stress, strengthening intestinal barrier function, and promoting the production of SCFAs (Li et al. 2024). Supplementation with probiotics has significantly reduced levels of tumor necrosis factor‐α (TNF‐α) and C‐reactive protein, as well as FPG, hemoglobin A_1c_ (HbA_1c_), and homeostatic model assessment for insulin resistance (HOMA‐IR) in T2DM patients, effectively improving their inflammatory status and glycemic control (Ding et al. 2021). Beyond SCFAs, other microbial metabolites, including BAs and amino acids, have also been implicated in modulating insulin sensitivity and metabolic homeostasis in T2DM (Gu et al. 2023; Liu et al. 2022). However, clinical evidence addressing the broader metabolic consequences of probiotic supplementation in T2DM remains limited.

Therefore, we conducted a randomized, double‐blind, placebo‐controlled trial to investigate the effects of a probiotic complex comprising *Bifidobacterium animalis* subsp. *lactis* BLa80, *Lacticaseibacillus rhamnosus* LRa05, and *Bifidobacterium breve* BBr60 on inflammatory responses, metabolic profiles, including BAs and amino acid metabolism, and gut microbiota composition in patients with T2DM. This study aims to provide clinical evidence supporting probiotic supplementation as an adjunctive approach to improve metabolic regulation and inflammation balance in T2DM management.

## 2 Materials and Methods

### 2.1 Study Design

This 12‐week, randomized, double‐blind, placebo‐controlled clinical trial was conducted at Nanchong Central Hospital (Nanchong, China) between April 2024 and May 2025. A total of 80 adult participants with T2DM were enrolled and randomly assigned (1:1) to receive either a multi‐strain probiotic formula or a matched placebo in addition to standard hypoglycemic therapy. The probiotic formula contained BLa80, LRa05, and BBr60, each provided at 10 billion colony‐forming units (CFU) per day (total 30 billion CFU/day). Randomization was computer‐generated, and both participants and investigators were blinded to group allocation throughout the study. The experimental protocol for this study secured official approval from the Ethics Committee of Nanchong Central Hospital (Ref: 2024‐Review [001]). Furthermore, the trial was formally listed on the https://https://clinicaltrials.gov registry under the identifier NCT06440486. All research activities and procedures conducted throughout the investigation rigorously adhered to the ethical principles outlined in the Declaration of Helsinki.

### 2.2 Participants

Prospective participants were considered for enrollment if they were between 18 and 75 years of age and had a clinically confirmed T2DM diagnosis. The key enrollment requirements included: (1) provision of written informed consent following a comprehensive explanation of the study's processes and associated risks; (2) age ≥ 18 years, any sex; (3) diagnosis of T2DM; and (4) absence of cardiovascular, renal, or other diabetic complications. Exclusion criteria were: (1) age > 75 years; (2) current insulin use or diabetic complications (e.g., cardiovascular disease); (3) concurrent treatment with α‐glucosidase inhibitors; (4) current or recent probiotic use; (5) pregnancy or lactation; and (6) any other condition deemed unsuitable by the investigators. All participants received both verbal and written explanations of the study and provided written informed consent prior to enrollment.

### 2.3 Sample Size Consideration

A formal sample size calculation was not performed for this preliminary, hypothesis‐generating study. The sample size of 80 participants was primarily determined based on practical constraints, including patient availability, resource allocation, and budgetary considerations over the study period. Furthermore, our sample size is comparable to those reported in several previous pilot and mechanistic studies investigating the effects of probiotics on gut microbiota and metabolic parameters in patients with T2DM (Ding et al. 2021; Ghoreishy et al. 2022; Feizollahzadeh et al. 2017). We acknowledge that the study may be underpowered to detect small but clinically significant effects in some of the exploratory outcomes, and this limitation is addressed in the Discussion section.

### 2.4 Randomization and Blinding

Eligible participants were randomized (1:1) to the probiotic or placebo group using R software (version 4.4.2) by an independent researcher not involved in the study. A simple randomization procedure was applied using a computer‐generated random number list. To safeguard against bias, allocation was concealed using sequentially numbered, opaque, sealed envelopes (SNOSE). The trial was conducted under double‐blind conditions, meaning that participants, investigators, clinical personnel, and outcome assessors remained fully blinded to the treatment assignments. To maintain blinding, probiotic and placebo products were identical in appearance, smell, and taste. The randomization code was retained by an independent third party and not revealed until all data analyses were completed.

### 2.5 Intervention

Participants in the probiotic group consumed one strip of the probiotic formulation daily for 12 weeks, in addition to standard hypoglycemic therapy. Each strip contained the probiotic mixture described above, with dextrin as the carrier. Participants in the placebo group received one matched strip per day containing only dextrin, identical in appearance, weight, color, and packaging to the probiotic product. Both products were supplied by Wecare Probiotic Co. Ltd. (Suzhou, China). Participants were instructed to consume the product once daily after breakfast with water. Compliance was assessed using self‐reported intake diaries and follow‐up visits every 4 weeks. Blood and fecal samples were collected at baseline and after 12 weeks of intervention and stored at −80°C until analysis.

### 2.6 Outcome Measurements

The primary endpoint of this study was the change from baseline in a panel of key inflammatory markers, including TNF‐α, interleukin‐4 (IL‐4), interleukin‐10 (IL‐10), interleukin‐17 (IL‐17). Key secondary endpoints included changes in glycemic parameters, host metabolic profiles, and gut microbiota composition. Specifically, glycemic parameters were assessed through FPG, total glycated hemoglobin (GHb), and HbA_1c_; host metabolic profiles were comprehensively evaluated via quantitative analysis of short‐chain fatty acids, comprehensive targeted profiling of bile acids, and quantitative analysis of serum amino acids; and gut microbiota composition was analyzed using 16S rRNA gene sequencing.

### 2.7 Serum Index Detection

HbA_1c_ (%) and GHb (%) were measured using high‐performance liquid chromatography with borate affinity chromatography. Quantification of the inflammatory mediators IL‐4, IL‐10, IL‐17, and TNF‐α was performed by flow cytometry, with concentrations expressed in pg/mL. The measurement of glucose levels was based on the hexokinase method and expressed in mmol/L. All tests were conducted strictly according to the hospital's standardized operating procedures.

### 2.8 Metabolic Index Detection

The analysis of metabolites was carried out using chromatographic‐mass spectrometric techniques. The quantitative profiling of 22 serum amino acids (Table S1) was conducted via Liquid Chromatography–Tandem Mass Spectrometry (LC–MS/MS). The assay was calibrated with isotopic internal reference standards (including Tryptophan‐d3), and all concentrations are reported in μg/mL. Concurrently, bile acid metabolic profiling was performed on the same LC–MS/MS platform. Meanwhile, short‐chain fatty acids (SCFAs) in fecal samples were analyzed by gas chromatography–mass spectrometry (GC–MS), employing 4‐methylvaleric acid as an internal standard for quantification. The SCFA concentrations are reported in μg/g of stool. For detailed methodologies, refer to Data S2.

### 2.9 DNA Extraction, and Sequencing

Extraction of total genomic DNA was carried out from the microbial community samples. Subsequently, its concentration and purity were assessed using a Nanodrop spectrophotometer, while integrity was verified by 1.2% agarose gel electrophoresis. Following this, specific gene regions were amplified via PCR with high‐fidelity DNA polymerase under rigorously optimized cycling parameters; negative controls were included to guarantee experimental integrity. The resultant PCR amplicons then underwent purification and recovery using magnetic beads, and their concentrations were determined by fluorescence quantification before being pooled as required for library construction. Next, sequencing libraries were prepared using Illumina's TruSeq Nano DNA LT Library Prep Kit, which included processes such as end repair, A‐tailing, adapter ligation, library enrichment, and purification, followed by library quality assessment and quantification. Finally, qualified libraries were subjected to high‐throughput sequencing using the MiSeq sequencer.

### 2.10 Bioinformatics Analysis

The raw sequencing data first underwent rigorous quality filtering, during which samples failing the quality thresholds were either re‐sequenced or supplemented. Demultiplexing was then performed based on index and barcode sequences, which were subsequently trimmed. Following this, amplicon sequence variants (ASVs) were generated through denoising, or operational taxonomic units (OTUs) were clustered using the QIIME2 DADA2 or VSEARCH pipelines. Alpha‐diversity, reflecting the microbial variation within individual samples, was calculated using a set of complementary indices: community richness was measured by the Chao1 index and observed species count; Shannon and Simpson indices were applied to evaluate community diversity; and Pielou's evenness (Pielou_e) quantified community uniformity. Statistical comparisons of these indices within groups (pre‐ versus post‐intervention) were conducted with the Wilcoxon signed‐rank test, while differences between the probiotic and placebo groups were assessed using the Mann–Whitney *U* test. A *p*‐value < 0.05 was considered statistically significant. Between‐sample microbial community structure (β‐diversity) was assessed based on Bray–Curtis dissimilarity. The results were visualized using principal coordinates analysis (PCoA). To visualize the results, we performed principal coordinates analysis (PCoA). The statistical significance of intergroup microbiota structural differences was tested for using permutational multivariate analysis of variance (PERMANOVA) with 999 permutations in the adonis2 function (R vegan package). To pinpoint bacterial taxa exhibiting significant differential abundance between the probiotic and placebo groups post‐intervention, we applied linear discriminant analysis effect size (LEfSe). For this analysis, an LDA score threshold of > 2.0 and a Kruskal–Wallis test alpha level of 0.05 were defined for identifying discriminative features.

### 2.11 Statistical Analysis

We presented continuous data exhibiting a normal distribution as mean ± standard deviation and analyzed them using the *t*‐test. Non‐normally distributed data were analyzed using non‐parametric tests the Mann–Whitney *U* test for comparisons between groups and the Wilcoxon signed‐rank test for within‐group longitudinal comparisons. For categorical data, relationships were tested with the Chi‐squared (*χ* ^*2*^) test or Fisher's exact test. The threshold for statistical significance was set at *p* < 0.05 for all primary analyses. In the metabolomics workflow, we filtered metabolites based on a detection rate exceeding 50% in the cohort. The significance of these metabolites was then evaluated by calculating *p*‐values via the *t*‐test or Mann–Whitney−Wilcoxon test for two‐group comparisons, and by one‐way ANOVA or the Kruskal–Wallis test for multi‐group comparisons. To ensure both statistical and model‐based relevance, a metabolite was considered significantly altered only if it met the dual criteria of a *p*‐value < 0.05 and a VIP score > 1. Given the exploratory nature of this study, no formal adjustment for multiple comparisons was applied, and all statistical tests were evaluated using a nominal two‐sided significance level of *p* < 0.05.

## 3 Results

### 3.1 Participant Characteristics

**FIGURE 1:**
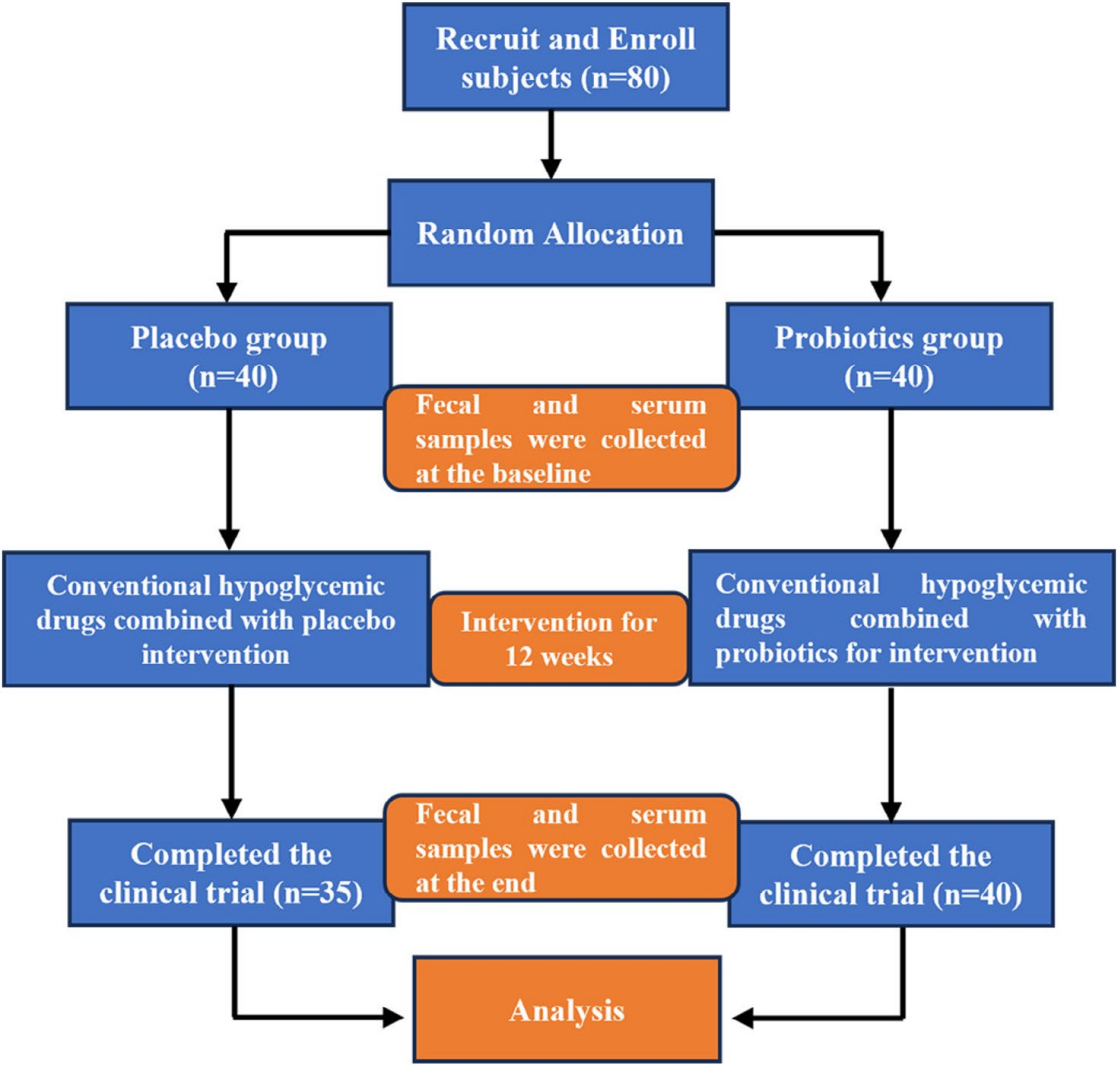
Presents a flowchart outlining participant recruitment and the study procedure.

A total of 80 participants were enrolled in this study. During the trial period, 5 participants withdrew from the research due to personal reasons. Post‐unblinding analysis revealed that all 5 withdrawn participants were from the placebo group. Ultimately, 75 participants completed the entire clinical trial (Figure 1). The probiotic intervention was well‐tolerated, with no serious adverse events reported. All withdrawals were documented as being due to personal reasons unrelated to the study intervention. The baseline characteristics of all participants are presented in Table 1. Statistical analysis showed no significant differences between the two groups in terms of gender distribution, age, or biochemical parameters (*p* > 0.05), demonstrating satisfactory comparability between the groups at baseline.

**TABLE 1:** Baseline characteristics of participants in the placebo and probiotic groups.

Characteristics	Placebo group (*n* = 35)	Probiotic group (*n* = 40)	*p*
Gender			0.3551
Male, *n* (%)	21 (60%)	19 (47.5%)	
Female, *n* (%)	14 (40%)	21 (52.5%)	
Age (Years)	61.14 ± 2.65	64.33 ± 1.96	0.5104
GHb (%)	13.48 ± 3.80	14.52 ± 3.52	0.3130
HbA_1c_ (%)	9.99 ± 2.35	10.24 ± 2.07	0.6652
FPG (mmol/L)	14.73 ± 4.62	15.98 ± 4.10	0.2925
GLU (mmol/L)	8.15 ± 1.99	8.67 ± 2.19	0.2728

*Note:* Categorical variables are presented as percentages and tested for significance using the chi‐square test. Normally distributed continuous data are reported as mean ± standard deviation and were evaluated by the *t*‐test.

Abbreviations: FPG, fasting plasma glucose; GHb, total glycated hemoglobin; GLU, glucose; HbA_1c_, hemoglobin A_1c_.

### 3.2 The Impact of Probiotic Intervention on Inflammation in T2DM Patients

**FIGURE 2:**
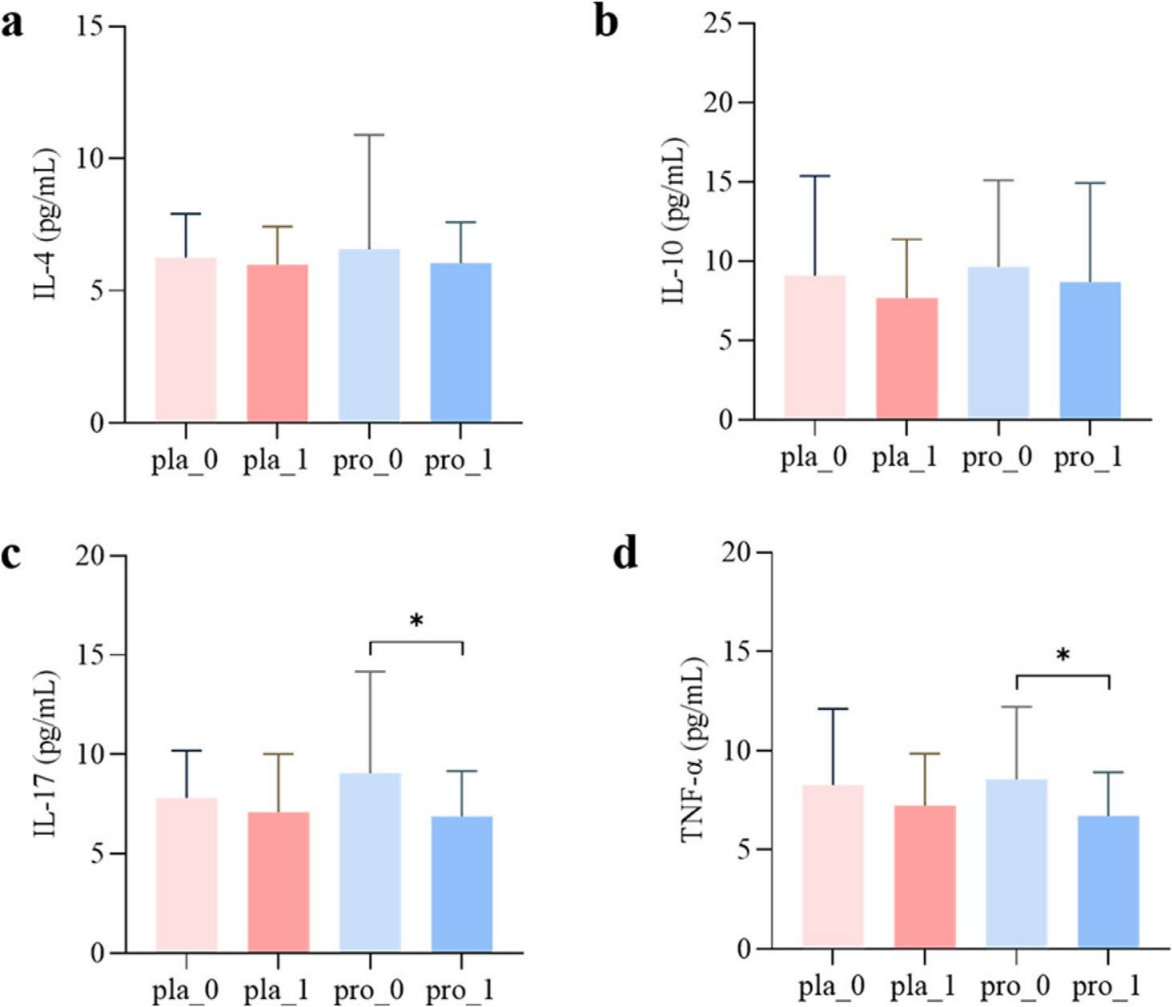
The impact of probiotic intervention on inflammatory factors in patients. (a) Levels of IL‐4 before and after intervention with placebo and probiotics; (b) Levels of IL‐10 before and after intervention with placebo and probiotics; (c) Levels of IL‐17 before and after intervention with placebo and probiotics; (d) Levels of TNF‐α before and after intervention with placebo and probiotics. *: *p* < 0.05, ns indicates no significant difference, pla_0, placebo group at baseline; pla_1, placebo group after 12 weeks of intervention; pro_0, probiotic group at baseline; pro_1, probiotic group after 12 weeks of intervention.

After probiotic intervention, the levels of IL‐17 and TNF‐α were significantly reduced (*p* < 0.05), while no significant changes were observed in the placebo group (*p* > 0.05) (Figure 2c,d). Additionally, no significant differences were found in the levels of IL‐4 and IL‐10 between the two groups (*p* > 0.05) (Figure 2a,b).

### 3.3 The Impact of Probiotic Intervention on Metabolic Function in Type 2 Diabetes Patients

Analysis of 22 serum amino acids in T2DM patients, who typically exhibit dysregulated metabolism marked by elevated concentrations of specific amino acids, revealed that a 12‐week probiotic intervention significantly reduced the circulating levels of threonine, isoleucine, and arginine compared to the placebo group (*p* < 0.05). This suggests a beneficial role for probiotics in ameliorating amino acid metabolic disturbances in T2DM (Table 2).

**TABLE 2:** Amino acids levels in patients after placebo and probiotic interventions.

Amino acids (μg/mL)	pla_1	pro_1	*p*
Gly	9.19 ± 2.31	10.08 ± 3.10	0.2122
Ala	11.87 ± 2.19	11.62 ± 2.16	0.6301
Ser	4.56 ± 0.55	4.64 ± 0.68	0.6814
Pro	7.86 ± 1.77	7.35 ± 1.69	0.2174
Val	13.17 ± 2.69	12.47 ± 3.24	0.2287
Thr	4.99 ± 0.80	4.68 ± 0.97	0.0424
Ile	6.79 ± 1.50	6.21 ± 1.75	0.0457
Leu	13.54 ± 2.69	12.82 ± 3.30	0.2160
Asn	2.67 ± 0.50	2.48 ± 0.56	0.1321
Orn	3.82 ± 0.90	3.86 ± 1.18	0.9417
Asp	0.74 ± 0.45	0.59 ± 0.22	0.1438
Hcy	0.17 ± 0.06	0.15 ± 0.04	0.1687
Gln	152.19 ± 18.91	152.40 ± 18.73	0.9412
Lys	9.84 ± 1.28	9.53 ± 1.55	0.3010
Glu	3.47 ± 1.04	3.56 ± 1.24	0.8909
Met	3.25 ± 0.36	3.11 ± 0.47	0.1258
His	4.72 ± 0.69	4.84 ± 0.64	0.4121
Phe	10.55 ± 1.48	10.01 ± 1.49	0.1208
Arg	4.22 ± 1.35	3.46 ± 0.71	0.0074
Tyr	10.30 ± 1.66	10.49 ± 1.79	0.6690
Trp	11.04 ± 2.26	10.72 ± 2.39	0.4905

*Note:* Data are presented as mean ± SD.

Abbreviations: Ala, alanine; Arg, arginine; Asn, asparagine; Asp, aspartic acid; Gln, glutamine; Glu, glutamic acid; Gly, glycine; Hcy, homocysteine; His, histidine; Ile, isoleucine; Leu, leucine; Lys, lysine; Met, methionine; Orn, ornithine; Phe, phenylalanine; Pla_1, placebo group after 12 weeks of intervention; Pro, proline; Pro_1, probiotic group after 12 weeks of intervention; Ser, serine; Thr, threonine; Trp, tryptophan; Tyr, tyrosine; Val, valine.

**FIGURE 3:**
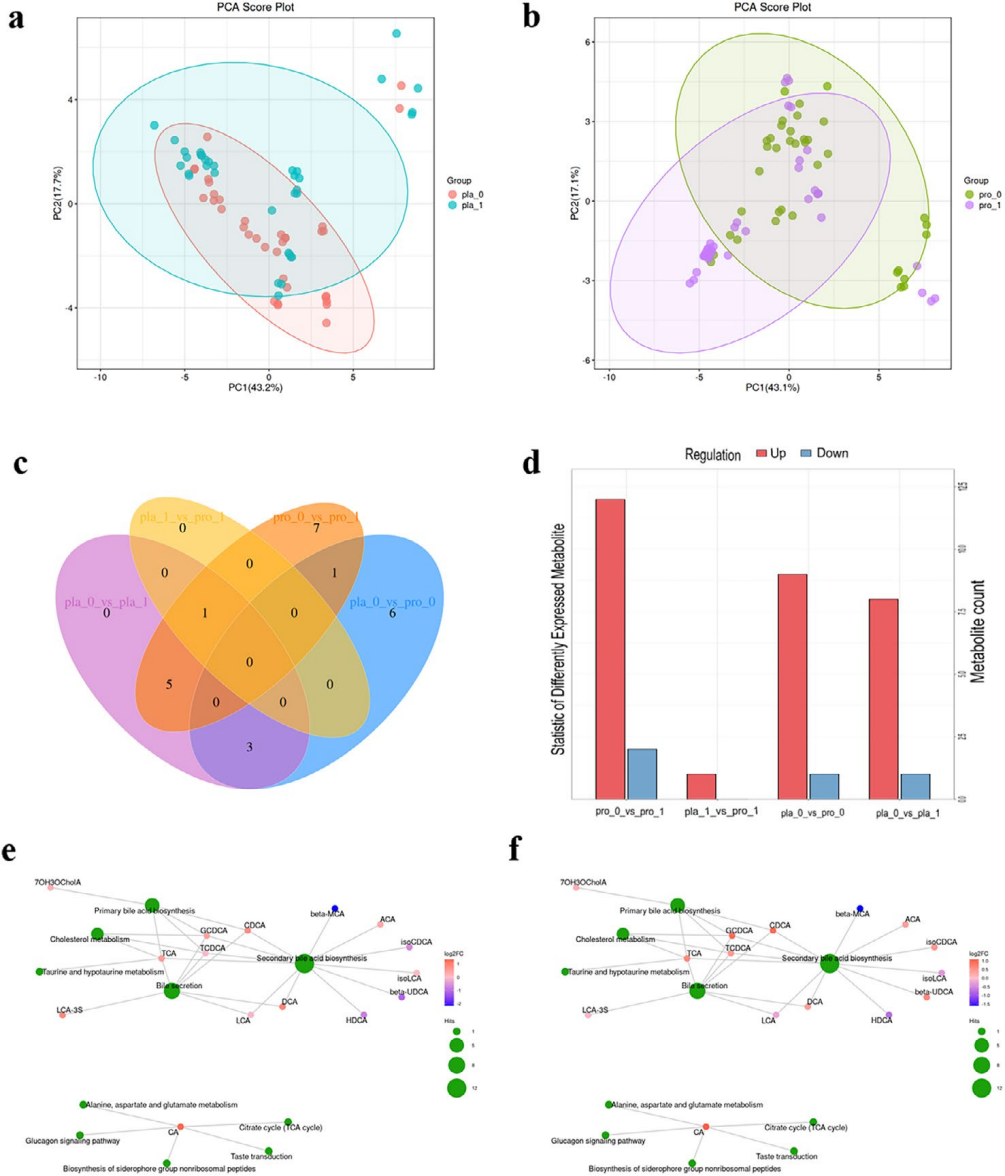
BAs metabolomics analysis. (a, b) Show PCA analysis at baseline and post‐intervention for the placebo and probiotic groups, respectively. (c) Presents a Venn diagram of differential metabolites between the placebo and probiotic groups. (d) Illustrates the count of differential metabolites between the placebo and probiotic groups. (e, f) Depict the differential metabolic pathway analysis at baseline and post‐intervention for the placebo and probiotic groups, respectively. Pla_0, placebo group at baseline; pla_1, placebo group after 12 weeks of intervention; pro_0, probiotic group at baseline; pro_1, probiotic group after 12 weeks of intervention.

Furthermore, we expanded our understanding of the mechanisms of action of probiotics by examining the serum BAs metabolites of participants (Figure 3). The results revealed that the probiotic intervention significantly altered the BA profile compared to pre‐intervention levels, with 16 BAs demonstrating significant changes. Among them, the levels of 7‐ketolithocholic acid (7‐ketoLCA), β‐muricholic acid (β‐MCA), and isolithocholic acid (isoLCA) increased significantly, while the levels of allocholic acid (ACA), β‐ursodeoxycholic acid (β‐UDCA), cholic acid (CA), and other BAs decreased significantly (Table S2). The impact of probiotic intervention on key metabolic pathways such as secondary BAs biosynthesis, bile secretion, and primary BAs biosynthesis was particularly pronounced, with the greatest changes in differential metabolites observed in these pathways (Table S3). Compared to the placebo group, the probiotic group showed significantly reduced levels of 7‐ketoLCA and β‐MCA, and the most significant changes in differential metabolites were found in key metabolic pathways like secondary BAs biosynthesis, bile secretion, and primary BAs biosynthesis (Tables S2 and S3).

**FIGURE 4:**
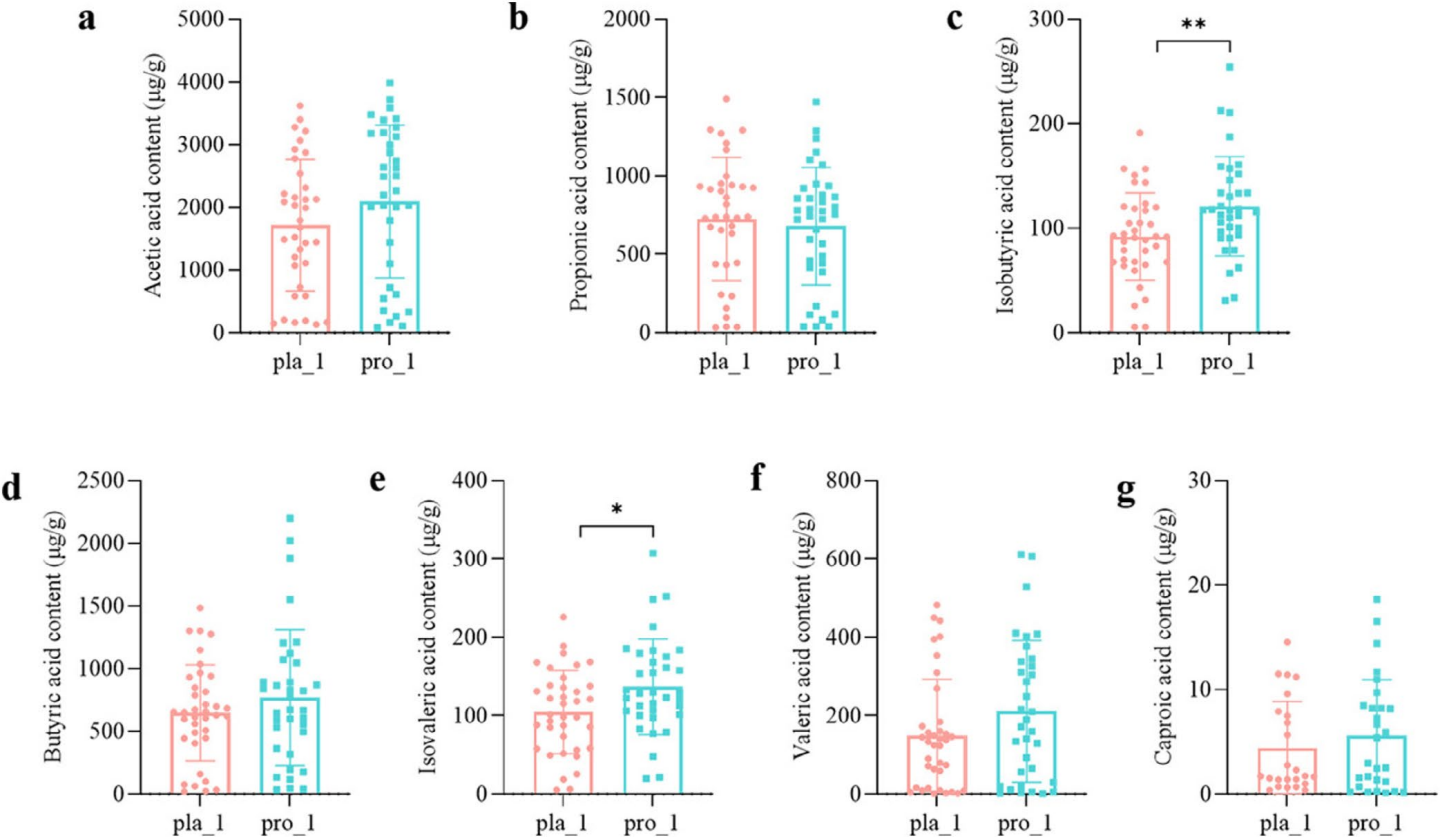
The impact of probiotic intervention on gut SCFAs in patients. (a–g) present the concentrations of acetic acid, propionic acid, isobutyric acid, butyric acid, isovaleric acid, valeric acid, and caproic acid in the gut following the 12‐week intervention in the two study groups. * *p* < 0.05, ** *p* < 0.01. _1: lacebo group after 12 weeks of intervention, _1: robiotic group after 12 weeks of intervention.

Given their physiological significance as key microbial metabolites, we focused on quantifying fecal short‐chain fatty acid (SCFA) concentrations in both patient cohorts, employing gas chromatography–mass spectrometry (GC–MS). The probiotic group displayed a marked increase in isobutyric and isovaleric acid levels relative to the placebo group (*p* < 0.05). A discernible upward trend was also observed for acetic acid, butyrate, valeric acid, and caproic acid in probiotic‐supplemented subjects (Figure 4).

### 3.4 The Impact of Probiotic Intervention on Glycemic Control in Patients With T2DM

**FIGURE 5:**
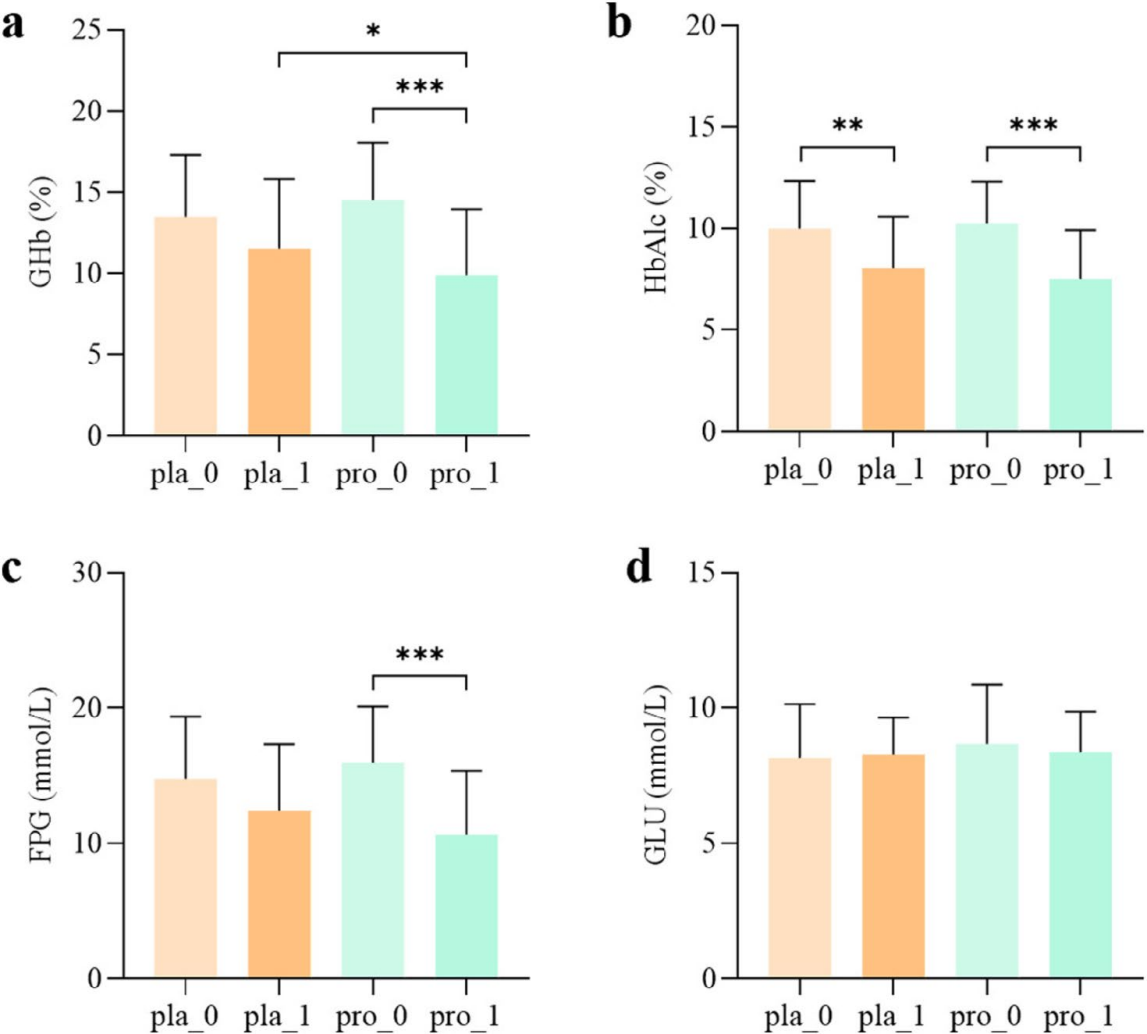
The impact of probiotic intervention on blood glucose control in patients. (a) Levels of GHb before and after intervention with placebo and probiotics; (b) Levels of glycated HbA_1c_ before and after intervention with placebo and probiotics; (c) Levels of fasting plasma glucose (FPG) before and after intervention with placebo and probiotics; (d) Levels of glucose (GLU) before and after intervention with placebo and probiotics. Pla_0, baseline placebo group; Pla_1, placebo group after 12 weeks of intervention; Pro_0, probiotic group at baseline; Pro_1, probiotic group after 12 weeks of intervention; GHb, total glycated hemoglobin; HbA_1c_, glycated hemoglobin A1c; FPG, fasting plasma glucose; GLU, glucose. * *p* < 0.05, ** *p* < 0.01, *** *p* < 0.001.

Probiotic intervention led to a significant decrease in both total glycated hemoglobin (GHb) and fasting plasma glucose (FPG) levels (*p* < 0.001). The final GHb value in the probiotic group was also significantly lower in comparison with the placebo group (*p* < 0.05; Figure 5a,c). Although hemoglobin A1c (HbA_1c_) levels decreased significantly in both cohorts after the 12‐week period (*p* < 0.01), intergroup analysis did not reach significance. No notable differences were detected in random glucose (GLU) levels between the two groups throughout the study (Figure 5b,d).

### 3.5 The Impact of Probiotic Intervention on Gut Microbiota in Type 2 Diabetes Patients

**FIGURE 6:**
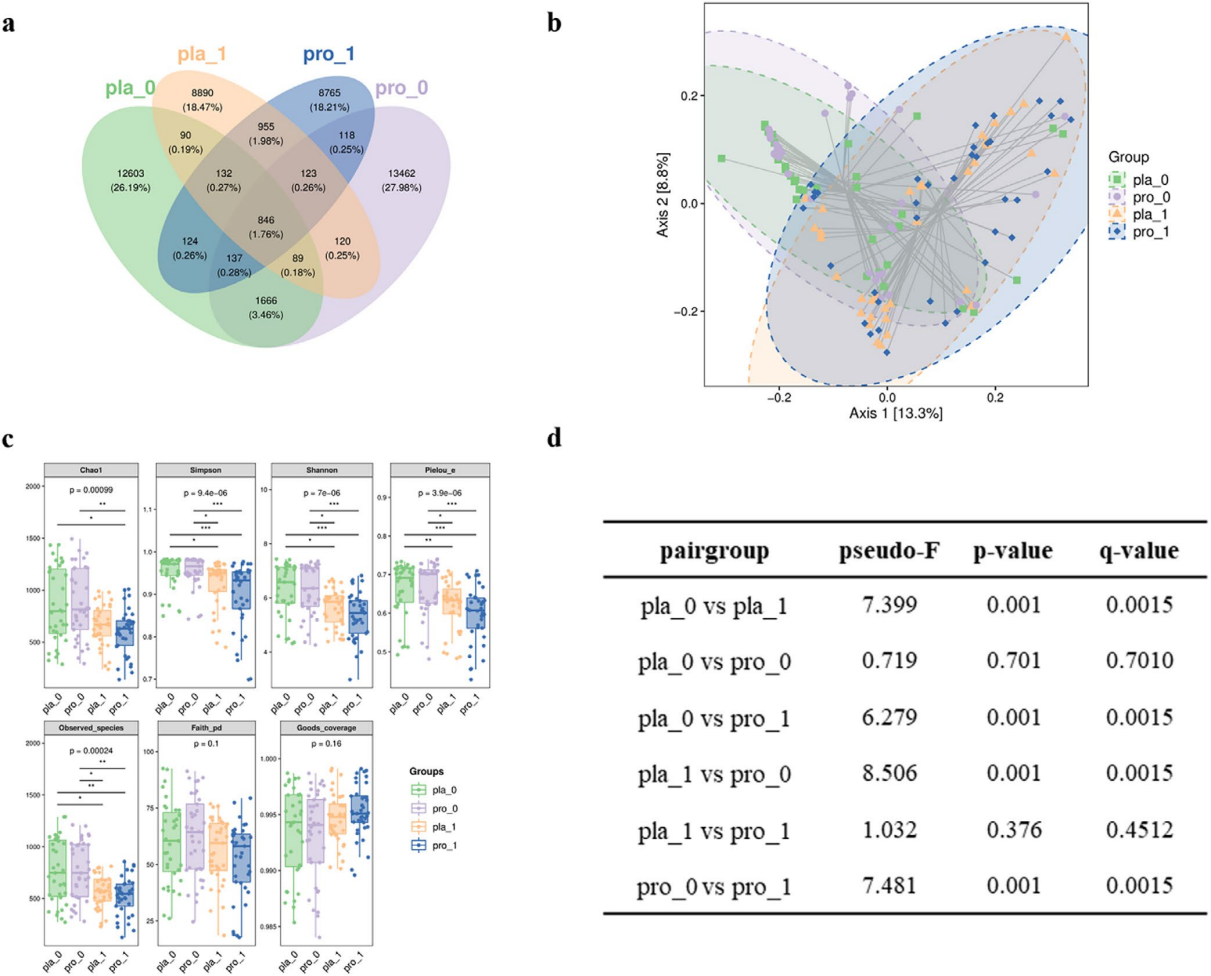
The alterations in gut microbiota alpha and beta diversity pre and postintervention. (a) A Venn diagram visualizes species distribution in the placebo and probiotic groups. (b) Beta diversity shifts postintervention are shown, based on principal component analysis of Bray Curtis distance among groups at different stages. Each colored point signifies a sample from a specific group. (c) The variation in alpha diversity after intervention is depicted. (d) Between group beta diversity differences are compared, with the *q*‐value reflecting the False Discovery Rate corrected *p*‐value.

Venn diagram analysis revealed that the placebo and probiotic groups shared 846 microbial species, accounting for only 1.76% of all detected species (Figure 6a), suggesting divergent microbial community structures between the two groups. Both experimental groups demonstrated a significant drop in multiple α‐diversity metrics by the end of the intervention. Specifically, the Chao1, Observed species, Shannon, Simpson, and Pielou_e indices all exhibited a significant reduction (*p* < 0.05), while Faith_pd and Good's coverage did not change significantly (Figure 6c). This reduction in α‐diversity likely reflects a selective modulation of the gut microbiota. β‐diversity analysis further indicated that the gut microbial structure significantly changed before and after intervention in both groups (Figure 6b,d) and (*p* = 0.0015), with intergroup comparisons showing no statistically significant differences in microbial structure between the placebo and probiotic groups at baseline or post‐intervention (*p* > 0.05).

Analysis of the gut microbiota at the phylum level revealed Firmicutes, Actinobacteriota, Proteobacteria, and Bacteroidota as the dominant phyla (Figure S1a). While some broad shifts in phylum abundances were observed following the intervention, such as an increase in Actinobacteriota and Proteobacteria in both groups, these high‐level changes are of limited functional specificity (Figure S1b–e). Therefore, we focused our subsequent analysis on the genus level to identify more taxonomically and mechanistically relevant alterations.

**FIGURE 7:**
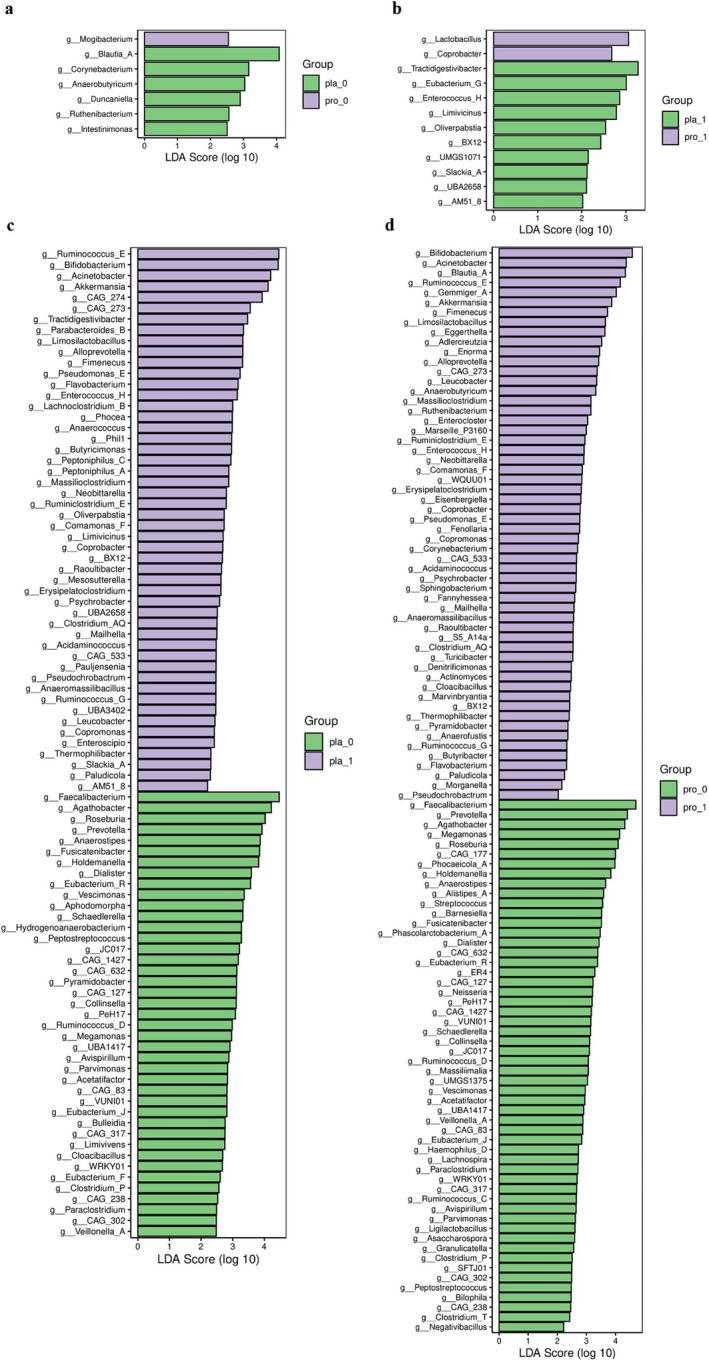
LEfSe analysis results of the genus‐level gut microbiota composition before and after intervention in the placebo and probiotic groups. (a) Changes at the genus level for the placebo group and probiotic group at baseline. (b) Changes at the genus level for the placebo group and probiotic group after intervention. (c) Changes in genus‐level composition before and after intervention for the placebo group. (d) Changes in genus‐level composition before and after intervention for the probiotic group. In panels (a–d), the vertical axis lists the discriminative taxonomic units, while the horizontal axis shows the corresponding LDA scores for each unit using bar graphs. Taxonomic units are sorted by their LDA scores, indicating their grouping specificity. The bar length corresponds to the magnitude of the difference, and the sample group exhibiting the highest abundance for each unit is indicated by the color.

The shifts observed at the genus level presented a more intricate pattern. While the probiotic group showed a notable elevation in the abundance of *Lactobacillus* and *Coprobacter* relative to the placebo (Figure 7b), the placebo group itself experienced a significant increase in *Coprobacter* from baseline to the end of the intervention (Figure 7c). After intervention, the abundance of *Bifidobacterium*, *Akkermansia*, and *Ruminococcus_E* increased significantly in both groups, while the abundance of *Peptostreptococcus* and *Collinsella* decreased significantly. It is noteworthy that the intervention specifically reduced the abundance of *Clostridium* in the probiotic group, with no such change observed in the placebo group (Figure 7c,d).

## 4 Discussion

Significant reductions in the pro‐inflammatory cytokines IL‐17 and TNF‐α were observed in T2DM patients following probiotic intervention. This finding aligns with results from several clinical meta‐analyses, reinforcing the systemic anti‐inflammatory potential of probiotics (Ding et al. 2021). Preclinical studies have reported that BLa80 promotes the proliferation of anti‐inflammatory cytokines and SCFA‐producing bacteria (Zhu et al. 2026), while BBr60 modulates inflammation‐related metabolic pathways (Wu et al. 2024; Bai et al. 2024). As pro‐inflammatory cytokines such as IL‐6 and TNF‐α can inhibit insulin signaling, impair glucose metabolism, and induce β‐cell apoptosis (Rehman et al. 2017; Wang et al. 2015), their suppression may help mitigate insulin resistance (Prattichizzo et al. 2018; Ehses et al. 2007). We therefore propose that the composite probiotic acts synergistically to attenuate inflammatory signaling cascades and remodel the intestinal environment, thereby contributing to improved metabolic homeostasis.

Amino acids, as fundamental components of proteins, are also critical signaling molecules for energy balance and metabolic homeostasis (Menni et al. 2013). Previous studies have indicated disordered amino acid metabolism in patients with T2DM, where elevated levels of certain amino acids, such as branched‐chain amino acids (e.g., isoleucine), histidine, and asparagine, are closely associated with insulin resistance and disease progression (Lai et al. 2020; Zhu et al. 2022). This study systematically evaluated the effects of probiotics on 22 serum amino acids and found that after 12 weeks of intervention, levels of threonine, isoleucine, and arginine were significantly reduced in the probiotic group compared to the placebo group (*p < 0.05*). This result has clear pathophysiological relevance: elevated threonine and isoleucine have been identified as risk markers for T2DM in multiple studies, and their reduction may reflect the modulation of metabolic disturbances by probiotics (Liu et al. 2023; Menni et al. 2013; Bloomgarden 2018; Ding et al. 2023). The alteration in arginine, a precursor for nitric oxide synthesis, might also be related to the improvement of vascular endothelial function in T2DM (Mahdi et al. 2018).

Regarding BAs, T2DM patients often exhibit dysregulation in the composition and proportion of BAs, such as decreased CDCA, increased CA and UDCA (Gao et al. 2022; Wu et al. 2020), and elevated levels of some taurine‐conjugated bile acids; these alterations are linked to disruptions in glucose and lipid homeostasis and impaired insulin sensitivity. (Lu et al. 2021; Doumatey et al. 2024; Zheng et al. 2021; Choucair et al. 2020). This study observed that probiotic intervention led to significant decreases in the levels of CA, UDCA, TCA, CDCA, and GHCA. The reduction in CDCA and GHCA is inconsistent with some previous reports, which might reflect strain‐specific regulatory effects of the probiotics or could be related to patient baseline characteristics, lifestyle, or disease heterogeneity. BAs metabolism involves a complex enterohepatic circulation and a microbiota co‐metabolism network, often resulting in multi‐directional changes.

Supplementing dietary fiber can promote the growth of specific SCFA‐producing bacteria. When these beneficial bacteria have greater diversity and abundance in the gut, patients exhibit better blood sugar control and more significant improvements in HbA_1c_ levels, partly achieved through increased GLP‐1 production, and also reduce the production of harmful metabolites. Restoring these SCFA‐producing bacteria may be a new strategy for managing T2DM (Zhao et al. 2018; Wang et al. 2020). In the present study, the probiotic group demonstrated a marked elevation in isobutyric and isovaleric acid levels relative to the placebo group. Furthermore, acetic acid, butyrate, valeric acid, and caproic acid contents also exhibited a clear upward trend. Substantial clinical evidence supports that adjunct probiotic use alongside conventional medication aids in glycemic management for T2DM patients (Ma et al. 2025). This study found that the composite probiotic intervention had a significant effect on blood sugar control in T2DM patients, with notably reduced levels of GHb, FPG, and HbA_1c_ after intervention in the probiotic group, consistent with previous research conclusions (Paquette et al. 2023). Although glycemic indices were not prespecified as primary endpoints, the observed reductions in fasting plasma glucose and glycated hemoglobin provide supportive evidence for the clinical relevance of the mechanistic findings.

Analysis of α‐diversity revealed a decline in richness (Chao1, Observed species), diversity (Shannon, Simpson), and evenness (Pielou_e) indices in both groups, while phylogenetic diversity (Faith_pd) and sequencing coverage (Good's coverage) remained stable. These results suggest that the intervention selectively simplified microbial community structure without disrupting its evolutionary foundation, possibly reflecting targeted regulation of functionally relevant taxa. β‐Diversity analysis indicated significant within‐group community shifts, but the overall intergroup difference remained modest, implying that probiotics exerted selective rather than global restructuring effects.

Previous studies have reported inconsistent findings regarding phylum‐level microbiota changes in T2DM (Larsen et al. 2010; Sedighi et al. 2017; Zhao et al. 2019). Our study also observed shifts in phylum abundance post‐intervention. However, given the limited functional resolution at this taxonomic level, we regard these as preliminary background data. Instead, the core of our discussion hinges on the genus‐level analysis, which reveals taxonomically precise changes that can be more directly linked to host metabolism. Additionally, the abundance of Bacteroidota significantly decreased after probiotic intervention, with a trend towards reduction also observed in the placebo group, although not significantly. After intervention with both the placebo and probiotics, the abundance of *Bifidobacterium*, *Akkermansia*, and *Ruminococcus_E* increased. Some studies have reported a significant decrease in the abundance of bifidobacteria in T2DM patients (Sedighi et al. 2017). *Akkermansia* plays a key role in maintaining the integrity of the mucus layer and reducing inflammation (Everard et al. 2013). Both *Bifidobacterium* and *Faecalibacterium* are beneficial bacteria associated with T2DM (Das and Gnanasambandan 2023; Wen et al. 2025). However, the abundance of *Faecalibacterium* significantly decreased before and after intervention in both groups. *Ruminococcus* and *Clostridium* are harmful bacteria related to T2DM (Das and Gnanasambandan 2023; Wen et al. 2025), but some studies also show a lack of butyrate‐producing microbial communities in T2DM patients, while *Ruminococcus* can promote the production of butyrate salts (Jiang et al. 2022). In this study, *Ruminococcus* showed taxonomic unit‐specific changes; although the abundance of *Ruminococcus_E/G* increased after intervention, *Ruminococcus_C/D* decreased. Placebo and probiotic interventions may improve dysbiosis by selectively regulating specific taxonomic units. After probiotic intervention, the abundance of Clostridium significantly decreased. In contrast, the placebo group showed no notable change, suggesting a beneficial role of probiotics in reducing this potentially harmful genus. Additionally, this study found that the abundance of *Collinsella* and *Peptostreptococcus* also significantly decreased after intervention, while previous studies have shown higher abundance of these genera in T2DM patients (Cunningham et al. 2021). This further confirms that probiotic intervention helps improve the dysbiosis of the gut microbiota in T2DM patients. Owing to the inherent complexity of gut microbiota composition, a comprehensive understanding of the mechanistic underpinnings of probiotic‐mediated regulation in T2DM remains to be fully established through future studies. However, given the complexity of the gut microbiota structure, more in‐depth mechanistic research is still required to fully explore the regulatory effects of probiotic intervention on the gut microbiota of T2DM patients.

We acknowledge that the probiotic formulation investigated was developed by a Chinese company, which may raise questions regarding the generalizability of our findings. However, it is important to note that the constituent strains BLa80, LRa05, and BBr60 possess a substantial international profile. First, these strains are commercially available in over 80 countries and regions worldwide, including markets in Europe (e.g., Spain, the Netherlands), South America (e.g., Brazil), and the Middle East (e.g., Turkey, Iran). Second, the core strains BLa80 and LRa05 have secured FDA GRAS certification, a recognized international safety standard. Third, their safety and biological activity have been further validated in independent clinical trials conducted in European populations. Therefore, the global availability, regulatory status, and existing clinical data for these strains strongly support the potential applicability of our metabolic and anti‐inflammatory findings to T2DM populations outside of China. Additionally, the review by Horwell et al. (Horwell et al. 2024) highlights infancy as a window of opportunity for microbial colonization and immune programming, with profound implications for long‐term metabolic health, including T2DM. This suggests that the roots of adult disease may lie in early life, and future research should explore early‐life microbiome interventions for diabetes prevention.

In summary, multi‐strain probiotic supplementation beneficially modulated inflammatory pathways, BAs, and gut microbial composition in patients with T2DM, providing mechanistic insight into its potential therapeutic utility. Several limitations warrant consideration. First, the sample size was based on feasibility rather than formal power analysis, which may limit statistical robustness. Second, owing to the exploratory nature of this study and the relatively limited sample size, multiple endpoints were analyzed without formal correction for multiple comparisons, which may increase the risk of false‐positive findings, particularly in metabolomics and microbiome analyses. Therefore, these results should be interpreted as hypothesis‐generating rather than confirmatory and warrant validation in larger, independent cohorts. Third, this study primarily focused on mechanistic endpoints such as inflammatory and metabolic markers rather than clinical outcomes or patient‐reported measures. Although these biomarkers are relevant to disease pathophysiology, the inclusion of health‐related quality of life assessments would strengthen clinical relevance. Future trials with larger samples, extended follow‐up, and integrated HRQL evaluations are needed to comprehensively elucidate the role of probiotics in T2DM management.

## 5 Conclusion

The composite probiotic intervention can significantly improve blood glucose control, reduce inflammatory factor levels, and regulate BA, SCFAs, and amino acid metabolism in T2DM patients. These findings suggest that probiotics may serve as an adjunct therapy in T2DM management, with a need for further research into their mechanisms of action and clinical application potential.

## Author Contributions

**Shuguang Fang:** writing – review and editing, project administration. **Sijia Zhu:** writing – review and editing, writing – original draft, visualization, formal analysis. **Yaojie Xiao:** writing – review and editing, sample collection. **Yan Qiao:** writing – original draft, writing – review and editing, investigation, experimental support. **Wenjing He:** data curation, writing – review and editing. **Kang Liu:** writing – review and editing, project administration, supervision. **Guiqin Song:** conceptualization, writing – review and editing.

## Funding

This work was supported by the Sichuan Science and Technology Program [2024NSFSC1973], the Nanchong Science and Technology Program [23JCYJPT0028], and the North Sichuan Medical College Science and Technology Program [CBY25‐QDA05, CBY24‐KP03].

## Ethics Statement

This study was approved by the Ethics Committee of Nanchong Central Hospital (ethical approval number 2024‐Review [001]) and registered on the ClinicalTrials.gov website (registration number NCT06440486).

## Consent

Informed consent was obtained from all participants included in the study.

## Conflicts of Interest

The authors declare no conflicts of interest.

## Supporting information


**Figure S1:** LEfSe analysis examined the gut microbiota composition changes at the phylum level in the placebo and probiotic groups before and after the intervention. (a) The distribution of gut microbial abundance at the phylum level across groups. (b) Between‐group differences in phylum‐level abundance between the placebo and probiotic groups at baseline. (c) Between‐group differences in phylum‐level abundance between the placebo and probiotic groups post‐intervention. (d) and (e) Within‐group differences in phylum‐level abundance before and after the intervention for the placebo and probiotic groups, respectively. In (b)–(e), the vertical axis lists taxa with significant group wise differences. The horizontal axis uses bar graphs to illustrate each taxonomic unit's LDA score. Taxonomic units, sorted by score values, show their role in sample grouping. Longer bars indicate more significant taxonomic unit differences, and the sample group with the greatest abundance for a given unit is coded by the bar color.

**Table S1:** Serum amino acid analysis.
**Table S2:** Differential metabolites analysis of bile acid metabolism.
**Table S3:** KEGG pathway analysis of bile acid metabolomics.

**Data S2:** Metabolica index detectioon.

## Data Availability

The data that supports the findings of this study are available in the Supporting Information of this article.
