# Supplementary material for: Multi‐Strain Probiotics BLa80, LRa05, and BBr60 Modulate Inflammation, Bile Acids, and Gut Microbiota in Type 2 Diabetes: A Randomized Controlled Trial
Source: Food Sci Nutr. 2026 Apr 10;14(4):e71735. doi: 10.1002/fsn3.71735 (PMC13066713; doi:10.1002/fsn3.71735)
Supplement: Supplementary file 1 — Figure S1: LEfSe analysis examined the gut microbiota composition changes at the phylum level in the placebo and probiotic groups before and after the intervention. (a) The distribution of gut microbial abundance at the phylum level across groups. (b) Between‐group differences in phylum‐level abundance between the placebo and probiotic groups at baseline. (c) Between‐group differences in phylum‐level abundance between the placebo and probiotic groups post‐intervention. (d) and (e) Within‐group differences in phylum‐level abundance before and after the intervention for the placebo and probiotic groups, respectively. In (b)–(e), the vertical axis lists taxa with significant group wise differences. The horizontal axis uses bar graphs to illustrate each taxonomic unit's LDA score. Taxonomic units, sorted by score values, show their role in sample grouping. Longer bars indicate more significant taxonomic unit differences, and the sample group with the greatest abundance for a given unit is coded by the bar color. [file FSN3-14-e71735-s003.docx]

**Figure S1**


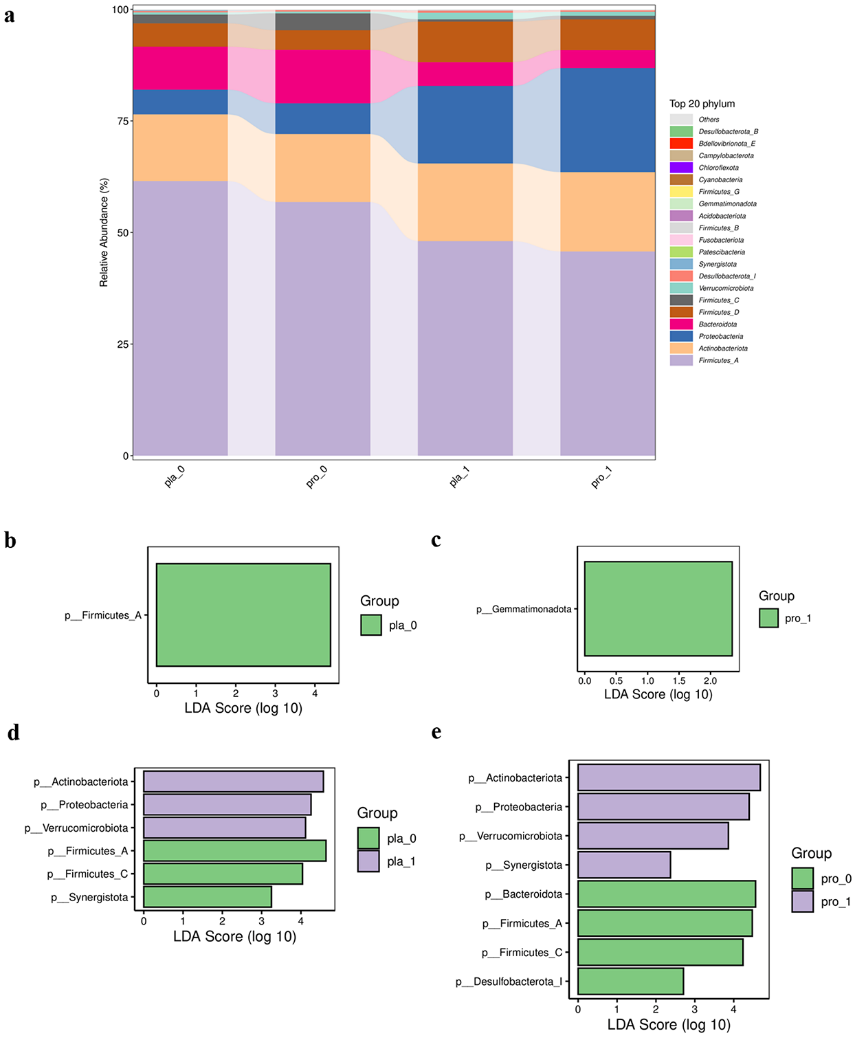


Figure S1 LEfSe analysis examined the gut microbiota composition changes at the phylum level in the placebo and probiotic groups before and after the intervention. (a) The distribution of gut microbial abundance at the phylum level across groups. (b) Between-group differences in phylum-level abundance between the placebo and probiotic groups at baseline. (c) Between-group differences in phylum-level abundance between the placebo and probiotic groups post-intervention. (d) and (e) Within-group differences in phylum-level abundance before and after the intervention for the placebo and probiotic groups, respectively. In (b) - (e), the vertical axis lists taxa with significant group - wise differences. The horizontal axis uses bar graphs to illustrate each taxonomic unit's LDA score. Taxonomic units, sorted by score values, show their role in sample grouping. Longer bars indicate more significant taxonomic unit differences, and the sample group with the greatest abundance for a given unit is coded by the bar color.
