# Supplementary material for: Multi‐Strain Probiotics BLa80, LRa05, and BBr60 Modulate Inflammation, Bile Acids, and Gut Microbiota in Type 2 Diabetes: A Randomized Controlled Trial
Source: Food Sci Nutr. 2026 Apr 10;14(4):e71735. doi: 10.1002/fsn3.71735 (PMC13066713; doi:10.1002/fsn3.71735)
Supplement: Supplementary file 3 — Data S2: Metabolica index detectioon. [file FSN3-14-e71735-s002.docx]

**Metabolic index detection**

**Amino acid metabolism**: This study employs Liquid Chromatography-Tandem Mass Spectrometry technology to detect 22 amino acid metabolites in serum (**Table S1**). Initially, individual standard solutions of amino acids are prepared using methanol or water, then mixed and diluted with 10% formic acid in methanol-water (1:1) to create working solutions. Simultaneously, an internal standard solution of Trp-d3 at 1000 ng/mL is prepared, and all solutions are stored refrigerated. For metabolite extraction, 100 μL of serum is mixed with 400 μL of 10% formic acid in methanol-water (1:1), vortexed for 30 seconds, and centrifuged at 12000 rpm and 4℃ for 5 minutes. The supernatant is diluted 10-fold, spiked with 20 ng/mL Trp-d3 internal standard, vortexed again for 30 seconds, and filtered through a 0.22 μm filter before being transferred to the injection vial (Fuertig et al., 2016; Virág et al., 2020). Chromatographic conditions include an ACQUITY UPLC® BEH C18 column (2.1×100 mm, 1.7 μm, Waters, USA), with an injection volume of 5 μL, column temperature at 40℃, mobile phase A (50% methanol in water with 0.1% formic acid) and B (10% methanol in water with 0.1% formic acid). The gradient elution is as follows: 0~6.5 min, 90~70% B; 6.5~7 min, 70~0% B; 7~14 min, 0% B; 14~14.5 min, 0~90% B; 14.5~17.5 min, 90% B. The flow rate is 0.3 mL/min from 0 to 8.0 min and 0.4 mL/min from 8.0 to 17.5 min. Mass spectrometry conditions include an electrospray ionization source in positive ion mode, with a source temperature of 500℃, voltage of 5500 V, collision gas at 6 psi, curtain gas at 30 psi, and nebulizer and auxiliary gases at 50 psi. Multiple reaction monitoring is used for scanning (Liyanaarachchi, Mahanama, Somasiri, & Punyasiri, 2018; Thiele, Stein, Oldiges, & Hofmann, 2012).

**Bile acid (BA)metabolism**: This study employs Liquid Chromatography-Tandem Mass Spectrometry technology to detect BAs metabolites in samples. BAs standards are accurately weighed and dissolved in methanol to prepare a mixed standard stock solution at a concentration of 1000 μg/mL, which is then diluted with 30% methanol to create 10 calibration curve points. For sample preparation, 100 μL of serum sample is placed in a 2 mL centrifuge tube, mixed with 600 μL of methanol at -20℃, vortexed for 60 seconds, and then centrifuged at 12000 rpm and 4℃ for 10 minutes. The supernatant (400 μL) is collected and concentrated to dryness using a vacuum concentrator. The residue is reconstituted in 100 μL of 30% methanol, filtered, and transferred to an injection vial for analysis (Bhargava et al., 2020). The chromatographic conditions include the use of an ACQUITY UPLC® BEH C18 column (2.1×100 mm, 1.7 μm, Waters, USA), with an injection volume of 5 μL, column temperature at 40℃, mobile phase A (0.01% formic acid in water), and mobile phase B (acetonitrile). Gradient elution is performed at a flow rate of 0.25 mL/min. The mass spectrometry conditions include an electrospray ionization (ESI) source in negative ion mode, with a source temperature of 500℃, voltage of -4500 V, collision gas at 6 psi, curtain gas at 30 psi, and nebulizer and auxiliary gases at 50 psi. Multiple reaction monitoring (MRM) is used for scanning (Hu, An, Shi, Li, & Liu, 2020; Yang et al., 2017).

**Short-chain fatty acid (SCFAs)**: This study uses gas Chromatography-mass spectrometry (GC-MS) to analyze SCFAs in fecal samples. Standards of Acetic, propionic acid, isobutyric, butyric, isovaleric, valeric caproic acids are prepared in water at 100 mg/mL and diluted to working solutions. Caproic acid standard is prepared in ether at 100 mg/mL and diluted similarly. The internal standard (4-methylvaleric acid) is prepared at 375 μg/mL in ether. For extraction, 50 mg of fecal sample is homogenized with 500 μL water and 100 mg glass beads for 1 min, then centrifuged at 12000 rpm and 4℃ for 10 min. The supernatant (200 μL) is mixed with 100 μL of 15% phosphoric acid, 20 μL internal standard, and 280 μL ether, homogenized for 1 min, and centrifuged again under the same conditions. The final supernatant is used for analysis (Zhang, Wang, & Zhu, 2019). Chromatographic conditions: Thermo Trace 1310 (Thermo Fisher Scientific, USA) system with Agilent HP-INNOWAX column (30 m × 0.25 mm × 0.25 μm), split injection (10:1), 1 μL injection volume, injector temperature 250℃, ion source temperature 300℃, transfer line temperature 250℃. Temperature program: 90℃ initial, ramp to 120℃ at 10℃/min, to 150℃ at 5℃/min, and to 250℃ at 25℃/min, hold for 2 min. Helium carrier gas at 1.0 mL/min. Mass spectrometry: Thermo ISQ 7000, EI source, 70 eV, SIM mode (Hsu et al., 2019).

**Reference**

Bhargava, P., Smith, M. D., Mische, L., Harrington, E., Fitzgerald, K. C., Martin, K., . . . Calabresi, P. A. (2020). Bile acid metabolism is altered in multiple sclerosis and supplementation ameliorates neuroinflammation. *The Journal of Clinical Investigation, 130*(7), 3467-3482. doi:10.1172/JCI129401

Fuertig, R., Ceci, A., Camus, S. M., Bezard, E., Luippold, A. H., & Hengerer, B. (2016). LC-MS/MS-based quantification of kynurenine metabolites, tryptophan, monoamines and neopterin in plasma, cerebrospinal fluid and brain. *Bioanalysis, 8*(18), 1903-1917. doi:10.4155/bio-2016-0111

Hsu, Y.-L., Chen, C.-C., Lin, Y.-T., Wu, W.-K., Chang, L.-C., Lai, C.-H., . . . Kuo, C.-H. (2019). Evaluation and Optimization of Sample Handling Methods for Quantification of Short-Chain Fatty Acids in Human Fecal Samples by GC-MS. *Journal of Proteome Research, 18*(5), 1948-1957. doi:10.1021/acs.jproteome.8b00536

Hu, T., An, Z., Shi, C., Li, P., & Liu, L. (2020). A sensitive and efficient method for simultaneous profiling of bile acids and fatty acids by UPLC-MS/MS. *Journal of Pharmaceutical and Biomedical Analysis, 178*, 112815. doi:10.1016/j.jpba.2019.112815

Liyanaarachchi, G. V. V., Mahanama, K. R. R., Somasiri, H. P. P. S., & Punyasiri, P. A. N. (2018). Development and validation of a method for direct, underivatized analysis of free amino acids in rice using liquid chromatography-tandem mass spectrometry. *Journal of Chromatography. A, 1568*, 131-139. doi:10.1016/j.chroma.2018.07.035

Thiele, B., Stein, N., Oldiges, M., & Hofmann, D. (2012). Direct analysis of underivatized amino acids in plant extracts by LC-MS-MS. *Methods In Molecular Biology (Clifton, N.J.), 828*, 317-328. doi:10.1007/978-1-61779-445-2_25

Virág, D., Király, M., Drahos, L., Édes, A. E., Gecse, K., Bagdy, G., . . . Ludányi, K. (2020). Development, validation and application of LC-MS/MS method for quantification of amino acids, kynurenine and serotonin in human plasma. *Journal of Pharmaceutical and Biomedical Analysis, 180*, 113018. doi:10.1016/j.jpba.2019.113018

Yang, T., Shu, T., Liu, G., Mei, H., Zhu, X., Huang, X., . . . Jiang, Z. (2017). Quantitative profiling of 19 bile acids in rat plasma, liver, bile and different intestinal section contents to investigate bile acid homeostasis and the application of temporal variation of endogenous bile acids. *The Journal of Steroid Biochemistry and Molecular Biology, 172*, 69-78. doi:10.1016/j.jsbmb.2017.05.015

Zhang, S., Wang, H., & Zhu, M.-J. (2019). A sensitive GC/MS detection method for analyzing microbial metabolites short chain fatty acids in fecal and serum samples. *Talanta, 196*, 249-254. doi:10.1016/j.talanta.2018.12.049
